# Beyond the Four-Level Model: Dark and Hot States in Quantum Dots Degrade Photonic Entanglement

**DOI:** 10.1021/acs.nanolett.2c04734

**Published:** 2023-02-06

**Authors:** Barbara Ursula Lehner, Tim Seidelmann, Gabriel Undeutsch, Christian Schimpf, Santanu Manna, Michał Gawełczyk, Saimon Filipe Covre da Silva, Xueyong Yuan, Sandra Stroj, Doris E. Reiter, Vollrath Martin Axt, Armando Rastelli

**Affiliations:** † Institute of Semiconductor and Solid State Physics, Johannes Kepler University, Linz4040, Austria; ‡ Secure and Correct Systems Lab, Linz Institute of Technology, 4040Linz, Austria; ¶ Theoretische Physik III, Universität Bayreuth, 95440Bayreuth, Germany; § Institute of Theoretical Physics, Faculty of Fundamental Problems of Technology, Wrocław University of Science and Technology, 50-370 Wrocław, Poland; ∥ School of Physics, Southeast University, Nanjing211189, China; ⊥ Forschungszentrum Mikrotechnik, FH Vorarlberg, 6850Dornbirn, Austria; # Condensed Matter Theory, TU Dortmund, 44221Dortmund, Germany

**Keywords:** Quantum Optics, Quantum Dots, Temperature Dependency, Excited States, Hot States, Entanglement

## Abstract

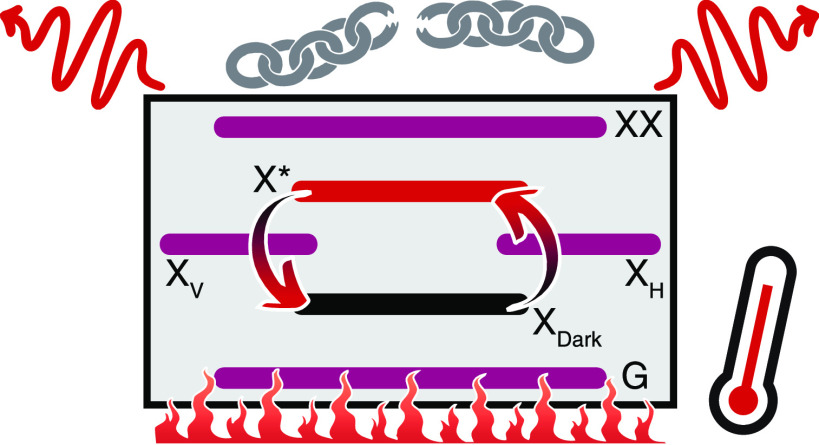

Entangled photon pairs are essential for a multitude of quantum photonic applications. To date, the best performing solid-state quantum emitters of entangled photons are semiconductor quantum dots operated around liquid-helium temperatures. To favor the widespread deployment of these sources, it is important to explore and understand their behavior at temperatures accessible with compact Stirling coolers. Here we study the polarization entanglement among photon pairs from the biexciton–exciton cascade in GaAs quantum dots at temperatures up to ∼65 K. We observe entanglement degradation accompanied by changes in decay dynamics, which we ascribe to thermal population and depopulation of hot and dark states in addition to the four levels relevant for photon pair generation. Detailed calculations considering the presence and characteristics of the additional states and phonon-assisted transitions support the interpretation. We expect these results to guide the optimization of quantum dots as sources of highly entangled photons at elevated temperatures.

Entangled photon pairs have been used to explore the validity of quantum mechanics and some of its least intuitive predictions.^1^ Besides being intriguing, entanglement is a key resource to establish correlations among remote locations, to achieve resolution beyond classical capabilities and for quantum information processing.^2−4^ In the last decades different methods have been developed to generate entangled photon pairs,^5^ such as parametric down-conversion,^6,7^ which has led to sources that can be operated in a wide temperature range^8^ and also in satellites.^9^ However, the stochastic photon generation process leads to an increase of the multipair generation probability and thus to a degradation of entanglement^10,11^ when the brightness is increased. In contrast, semiconductor quantum dots (QDs)^12^ are quantum emitters exhibiting sub-Poissonian emission characteristics and ultralow multiphoton pair emission probability even at maximum brightness. As a consequence, it may become possible for QDs, e.g., to outperform the secure key rate achievable with probabilistic sources in entanglement-based quantum-key-distribution.^13^ In ideal QDs, a polarization-entangled photon pair can be obtained by initializing the system in a biexciton |*XX*⟩ state,^14,15^ which decays back to the crystal ground state |*G*⟩ via two bright and energy-degenerate excitonic |*X*_*H*/*V*_⟩ states following two possible decay paths [inset of Figure 1(b)]. In particular, GaAs QDs obtained via the local droplet etching (LDE) method in an AlGaAs matrix^16−20^ have demonstrated excellent performance as sources of single entangled-photon pairs with a fidelity to one of the maximally entangled Bell states as high as 0.98.^21,22^ Thus far, the best results have been obtained at cryogenic temperatures, reachable with liquid-helium-based (wet) cryostats or bulky and energy-intensive closed-cycle (dry) cryostats. To achieve further advances with QD light sources, possibly enabling their deployment in space applications with light and energy-efficient cryo-coolers,^23^ a comprehensive study of entanglement at different operation temperatures *T* is needed. By using strain-tunable GaAs QDs capable of generating nearly perfectly entangled photons at low temperatures,^21^ we investigate the effects produced by increasing *T* on the entanglement and on the exciton decay dynamics following coherent |*XX*⟩ excitation. While the multipair emission probability remains low for all investigated temperatures, we find that entanglement degrades for *T* ≳ 15 K, which is accompanied by a slowed excitonic decay and weak light emission from higher-energy (“hot”) excitonic states. To gain insight into this rich evolution, we (i) expand the four-level model for the biexciton–exciton cascade by including hot and dark excitonic states, (ii) address the properties of such additional levels as well as of the corresponding radiative and nonradiative transitions by experiments and 8-band *k*·*p* and configuration-interaction (CI) calculations, (iii) model the population dynamics with the Liouville–von Neumann equation with Lindblad terms and rate equations, and (iv) evaluate the two-time correlation functions and degree of entanglement based on density matrix methods. Our calculations reproduce very well the experimental results and provide evidence that both the degradation of entanglement and the changes in decay dynamics for the |*XX*⟩ → |*X*_*H*/*V*_⟩ (shortly XX) and |*X*_*H*/*V*_⟩ → |*G*⟩ (X) transitions can be traced back to the thermal population and depopulation of excited and dark exciton states and to spin mixing.

**Figure 1:**
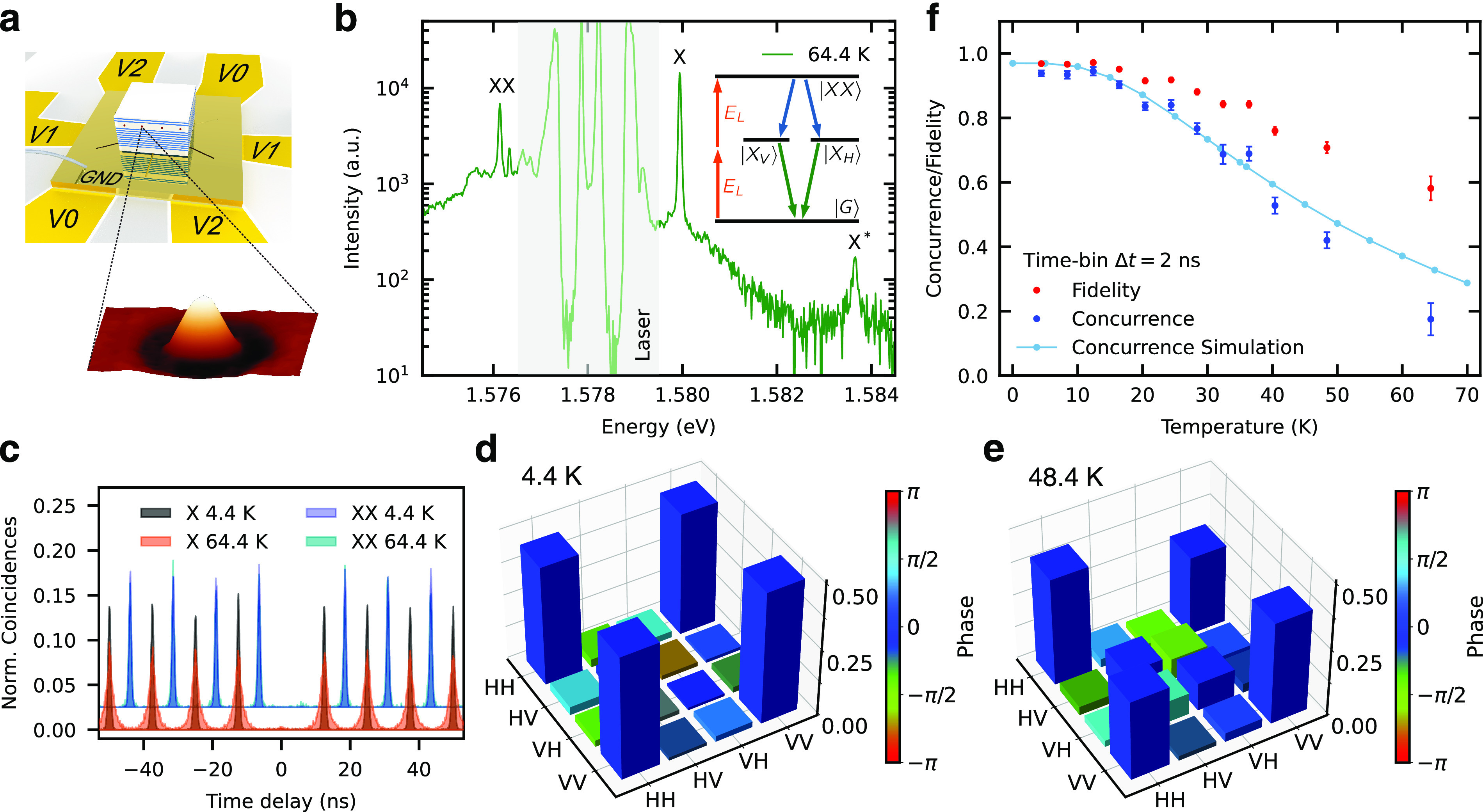
Measurements on a GaAs quantum dot at temperatures *T* up to about 65 K. (a) Sketch of the strain-tuning device with the sample containing the QDs and 150 × 150 × 9 nm^3^ atomic force microscopy image of a GaAs QD. (b) Photoluminescence spectrum under resonant two-photon excitation at nominal 64.4 K. Besides the exciton (X) and biexciton (XX), radiative transitions of higher-energy excitonic states X* become visible. The shaded area marks the laser stray light. Inset: The excitation scheme showing the laser energy *E*_*L*_ and the basic 4-level model including biexciton |*XX*⟩, bright excitons |*X*_*H*/*V*_⟩, and the ground state |*G*⟩. (c) *g*^(2)^ autocorrelation measurements of X and XX at 4.4 and 64.4 K. (d) and (e) Two-photon density matrices reconstructed from polarization-resolved cross-correlation measurements at 4.4 and 48.4 K. (f) Measured concurrence (blue dots) and fidelity (red dots) by using a 2 ns time bin. The theoretical simulation of the concurrence is shown in light blue.

GaAs QDs grown via the LDE method can be optimized to have |*X*_*H*/*V*_⟩ almost degenerate.^16,17^ Nevertheless, a finite energy splitting (or fine-structure splitting, FSS) generally remains, leading to a time evolution of the entangled state and consequent entanglement degradation in time-integrated measurements.^24^ Therefore, we make use of a piezoelectric strain-tuning device^21,25^ to cancel the FSS; see Figure 1(a). For the optical excitation of an individual QD, we use resonant two-photon excitation (TPE) [inset of Figure 1(b)] by tuning the energy *E*_*L*_ of a pulsed laser with a 80 MHz repetition rate to half of the difference between the |*XX*⟩ and |*G*⟩ states^26,27^ and by setting the laser power to obtain the maximum XX intensity (π-pulse conditions). The recorded photoluminescence (PL) spectrum under TPE at *T* = 64.4 K is shown in Figure 1(b). Besides the XX at 1.5761 eV and X at 1.5799 eV, a weaker line at 1.5836 eV is also visible, which we denote as X* and attribute to a thermally populated excitonic state. Spectra collected at different temperatures, showing also further excited states, can be found in the Supporting Information.^28^

To assess the effect of *T* on the light emission characteristics and entangled photon generation following optical excitation, we performed our study by increasing *T* stepwise, in a range from 4.4 to 64.4 K. For each *T* value we canceled the FSS via strain-tuning. First, the *g*^(2)^ autocorrelation functions of both the XX and X were recorded. In Figure 1(c) a clear broadening of the X histogram peaks is visible at *T* = 64.4 K compared to low-temperature data. In addition, a slight increase in *g*^(2)^(0) (see Figure 3 Supporting Information(28)) for XX and X from *g*_XX_^(2)^(0) = 0.008(1) to *g*_XX_^(2)^(0) = 0.032(5) and from *g*_X_^(2)^(0) = 0.008(1) to *g*_X_^(2)^(0) = 0.033(3) is visible.

A full state tomography was performed to obtain the two-qubit density matrices in polarization space, using a maximum likelihood method.^29^ Two representative density matrices for 4.4 and 48.4 K are shown in Figures 1(d) and (e). For higher temperatures, we observe decreasing VV-HH coherence with respect to HH-HH and VV-VV occupations, as well as rising HV and VH elements, indicative of state mixing. We evaluate concurrence and fidelity at every temperature for a time bin of 2 ns, as shown in Figure 1(f). At low temperatures the concurrence [fidelity] is equal to 0.94(1) [0.969(4)], comparable to former investigations.^21,30,31^ The degree of entanglement stays approximately constant up to about 15 K and then decreases with increasing temperature. The slight increase in *g*^(2)^(0) mentioned above does not explain the observed steep entanglement degradation shown in Figures 1(d–f) since the *g*^(2)^(0) values are still in the range typically observed at *T* = 5 K.^30,32,33^

To gain further insights into the origin of the entanglement degradation, we study the decay dynamics of the XX and X emission following excitation as shown in Figure 2. The upper half of Figure 2(a) shows the normalized measured decay traces for all explored temperatures. At *T* = 4.4 K, the XX emission shows an exponential decay, from which we extract a XX lifetime of 129(3) ps. With increasing *T* the XX decay first shows an increased slope and then becomes slower at longer time scales, resulting in a crossing of the 4.4 K and the 64.4 K decay traces at around 0.6 ns. A much stronger *T*-dependence is observed for the X decay: At *T* = 4.4 K the X intensity first rises and then exponentially decays with a time constant of 231(4) ps, as expected for a cascaded decay. For increasing *T* we see a slightly accelerated decay at short time scales similar to XX and—most importantly—an increasingly pronounced tail for long time scales, reminiscent of the slow decay observed under excited-state excitation at low temperatures.^32,34^ To understand this behavior, we extend the exciton-decay model of ref (35) to the biexciton decay. On top of the usual four levels shown in the inset of Figure 1(b), we add two dark exciton states |*X*_*D*_⟩, as well as excited states of the exciton |X*⟩ and biexciton |XX*⟩; see Figure 2(b). In the single-particle picture, the “hot” |XX*⟩ and |X*⟩ states are configurations where the electrons are in the “*s*-shell” and the holes in the “*p*-shell”. The energy difference *ΔE* = 3.7 meV between |*X*⟩ and |X*⟩ is taken from the recorded spectrum in Figure 1(b). Because |X*⟩ consists of an electron and a hole, four spin configurations are possible, resulting in four possible transitions. For purely heavy-hole excitons we would expect two bright and two dark states, similar to the ground-state exciton. In ref (36) a triplet was instead observed, which we ascribe to the high light-hole contribution of almost 40% (with ∼25% of bright admixture) to “*p*-shell” holes.^36−38^ As we are not interested in the detailed population of the excited states, we include |X*⟩ as a single state with multiplicity of four. For the |XX*⟩, we expect two “*s*-shell” electrons in a singlet state and two holes, one in the “*s*-shell” and the other in the “*p*-shell”, resulting again in four possible configurations. Since we were not able to unequivocally identify the emission lines associated with |XX*⟩, we assume the same value of 3.7 meV for the |*XX*⟩ – |XX*⟩ energy separation. Finally for the bright-dark splitting we take a value of 110 μeV, as in ref (36), and a multiplicity of two. We note that excited states with the electron in the “*p*-shell” are unlikely to be populated in the explored temperature range due to significantly higher energy differences of 15–20 meV according to our calculation (see Supporting Information(28)). States with holes in higher-energy shells play instead a role for *T* ≳ 40 K (see below) but are omitted from our model for the sake of simplicity.

**Figure 2:**
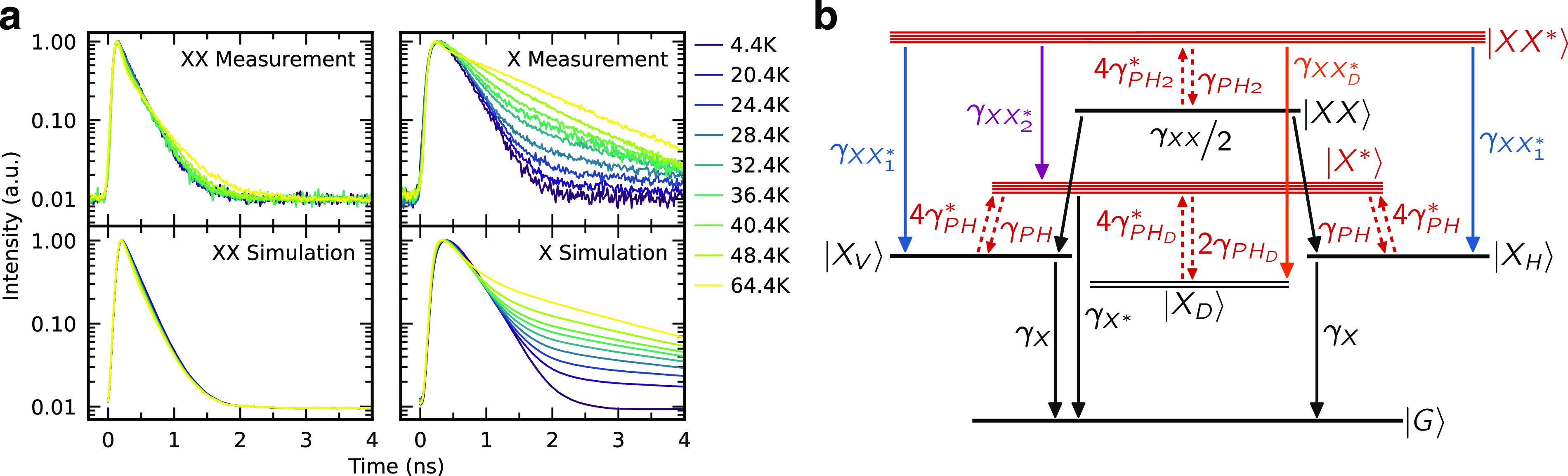
(a) Measurement and simulation of biexciton (XX) and exciton (X) decay dynamics at temperatures from 4.4 to 64.4 K. (b) Sketch of the energy levels used to model the decay dynamics. Excited biexciton and exciton states (|XX*⟩ and |X*⟩) are shown in red, while the four levels relevant for the generation of entangled photon pairs as well as the dark excitonic states are shown in black. γ_*X*_ (γ_*XX*_) is the rate associated with the radiative decay of the |*X*⟩ (|*XX*⟩) states. Temperature-dependent transitions due to phonon emission and absorption processes are shown as dashed red arrows, whereby the prefactors correspond to the multiplicity of the |XX*⟩, |X*⟩ and the dark exciton |*X*_*D*_⟩ states, assumed energetically degenerate. The rates of the shown transitions can be found in Table 1.

With reference to the levels shown in Figure 2(b) we now focus on the radiative recombinations (solid lines) and nonradiative phonon-assisted transitions (dashed lines) and their rates. Different from the dominant XX and X emission lines, which are characterized by the rates γ_*X*_ and γ_*XX*_, the recombination rate γ_X_^*^ of |X*⟩ is relatively weak because of the different envelope function symmetry for the electron and hole, but clearly visible at high temperature [Figure 1(b)], under nonresonant excitation,^36^ and in PL-excitation measurements.^32,39^ For radiative recombinations involving the same single-particle states we assume the same values for the corresponding rates. As an example, the recombination of |XX*⟩ leaving the system in a ground-state exciton |*X*_*H*/*V*_⟩ (|*X*_*D*_⟩) takes place with a rate γ_*XX*_1_^*^_ (γ_*XX*_D_^*^_) with γ_*XX*_1_^*^_ = γ_*XX*_D_^*^_ = γ_X_*, since in all cases we have a “*p*-shell” hole recombining with an “*s*-shell” electron. Further, the rate γ_*XX*_2_^*^_ for the |XX*⟩ recombination leaving the system in the |X*⟩ state is assumed to be equal to γ_*X*_ since an “*s*-shell” electron recombines with an “*s*-shell” hole.

For the phonon-assisted transitions we assume a *T*-dependence that is determined by the expected phonon number according to the Bose–Einstein distribution:
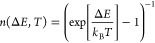
1where *k*_B_ is the Boltzmann constant. For *T* ≃ 43 K, *k*_B_*T* is comparable to *ΔE*, making the occupation of hole-dominated hot states likely. |X*⟩ relaxes to |*X*_*V*/*H*_⟩ with a rate
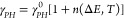
2where γ_*PH*_^0^ = 1 ns^–1^ is the phonon-assisted relaxation rate at low temperature, estimated from simulations (see section “Phonon-assisted relaxation” of the Supporting Information(28)) and fully consistent with the slow relaxation previously reported in refs (32) and (34) for similar QDs. |X*⟩ is populated via the phonon-mediated rate 4γ_PH_^*^, where the factor 4 in this and other rates corresponds to the state multiplicity discussed above, with
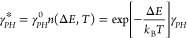
3

To justify the assumption of equal phonon-assisted rates for transitions involving states with different spin configurations and to fully understand the entanglement degradation observed in Figure 1(f), it is important to note that the highly mixed character of excited hole levels implies that the spin projection along the growth axis is not a good quantum number for the hot states. As a consequence, phonon-assisted relaxations are effectively not spin-conserving (see Supporting Information). This is in good agreement with PL-excitation measurements, where the |X*⟩ was excited resonantly and equal intensities for the *X*_*V*_ and *X*_*H*_ transitions independent of laser polarization were observed after relaxation. Calculations also confirm this almost “spin-agnostic” relaxation from all four |X*⟩ to all bright and dark |*X*⟩ states with at most 40% difference in rates (see Supporting Information), which justifies taking them equal for the sake of simplicity. The same approach is followed for the transitions involving |XX*⟩. Note that spin-flips between bright and dark exciton states are neglected since they are expected to occur at time scales in the order of μs.^35^ All rates used in our model and the corresponding origins are summarized in Table 1.

**Table 1:** Rates of the Decay Model Used for Theoretical Calculations

**Rate**	**in ns**^**-1**^
γ_*X*_	1/0.231(4)^a^
γ_X*_	1/10^b^
γ_*XX*_	1/0.129(3)^a^
γ_*XX*_D_^*^_	= γ_X*_
γ_*XX*_1_^*^_	= γ_X*_
γ_*XX*_2_^*^_	= γ_*X*_
γ_*PH*_^0^	1^c^

a Values taken from the PL measurements following TPE excitation at 4.4 K.

b Value estimated from the comparison between the results of the rate equation model and PL intensities of the X* line as well as from the *k*·*p* and CI simulations (see Supporting Information(28)).

c Values estimated from the *k*·*p* and CI simulations.

Solving the rate equations for the presented system and convolving the obtained time evolutions with a measured instrument response function result in the decay traces shown in the lower panels of Figure 2(a). The simulation reproduces both the initial acceleration of the decay observed for the XX and X—due to population loss through thermal excitation of the hot states—and the pronounced slow decay of the X signal—due to the repopulation of the bright |*X*_*V*/*H*_⟩ states. As in the experiment, an increasing *T* mostly affects the X dynamics. From the model, the slowed X decay is mostly produced by the slow phonon-assisted relaxation rate γ_*PH*_^0^ compared to γ_*X*_ combined with the state multiplicity of the |X*⟩ state, which thus acts as a reservoir slowly feeding the population of the |*X*_*V*/*H*_⟩ states. We notice that, for *T* ≳ 40 K, the measured X decay is still slower than the predicted decay, which we attribute to additional phonon-assisted population of higher-energy states, that are not taken into account in the presented model (see Supporting Information).

We now turn to the effect of the thermally induced processes described above on the degree of entanglement of XX-X photon pairs. To this end, numerical simulations are performed, solving the corresponding Liouville–von Neumann equation. In a first step, the photonic two-qubit density matrix is theoretically calculated based on polarization-resolved, time-integrated two-time correlation functions,^40^ modeling the experimental measurements. Afterward the simulated concurrence is directly evaluated from the obtained photonic density matrices. The result of these simulations is shown in Figure 1(f) in light blue for temperatures from 0 to 70 K. Note that the simulations do not predict a unity concurrence for low temperatures even for zero FSS, since the TPE sets a limit to the obtainable degree of entanglement due to a dynamic Stark shift of one exciton level induced by the excitation itself.^40^ The simulated result reproduces well the concurrence plateau at lower temperatures followed by a decrease starting around 16 K. Again, theory and experiment are in good agreement up to *T* = 40 K. For higher temperatures, the theory predicts a slower degradation compared to the experiment, consistent to additional phonon-assisted excitation channels.

In addition to the concurrence calculations, we use the decay model shown in Figure 2(b) to compute the photonic two-qubit density matrices and compare them with the experimentally reconstructed matrices, where mixing and decoherence emerge with increasing *T*; see Figure 1(e). In Figure 3(a) a representative histogram of the difference between the detection times of X and XX photons at *T* = 32.4 K is shown, displaying on the right side of the peaks coincidences that arise from the slow X decay. Coincidences within the chosen time bin of 500 ps (black dashed lines), corresponding approximately to our detector resolution are summed up and compared with the average area of the side peaks in the same interval. Figure 3(b) shows the measured and simulated real part of the density matrices for this time bin. The leftmost 2D diagrams in the gray and orange boxes in Figure 3(c) correspond to the 3D representations in Figure 3(b). Next, we begin to shift the time bin to higher time delays, indicated by the blue arrows in Figure 3(a) and above Figure 3(c). The resulting density matrices for a time delay up to 1050 ps are shown in panel (c). The gray box shows the measurement, the orange box the density matrices obtained from corresponding simulations. In order to mimic the time-filtering analysis theoretically, only photon pairs with a delay time in the respective interval/time bin are considered in the time-integrated correlation functions; cf., Supporting Information. Both experiment and theory show increasing state mixing (rise of HV-HV and VH-VH elements) and decoherence (drop of HH-VV and VV-HH elements) with increasing time delay. In turn, this finding is consistent with our dynamic model, in which the detection events producing the “tail” in the coincidence histograms stem from thermal cycling among levels, i.e. the occupation of hot and dark states at elevated temperatures followed by bright exciton repopulation with no spin memory.

**Figure 3:**
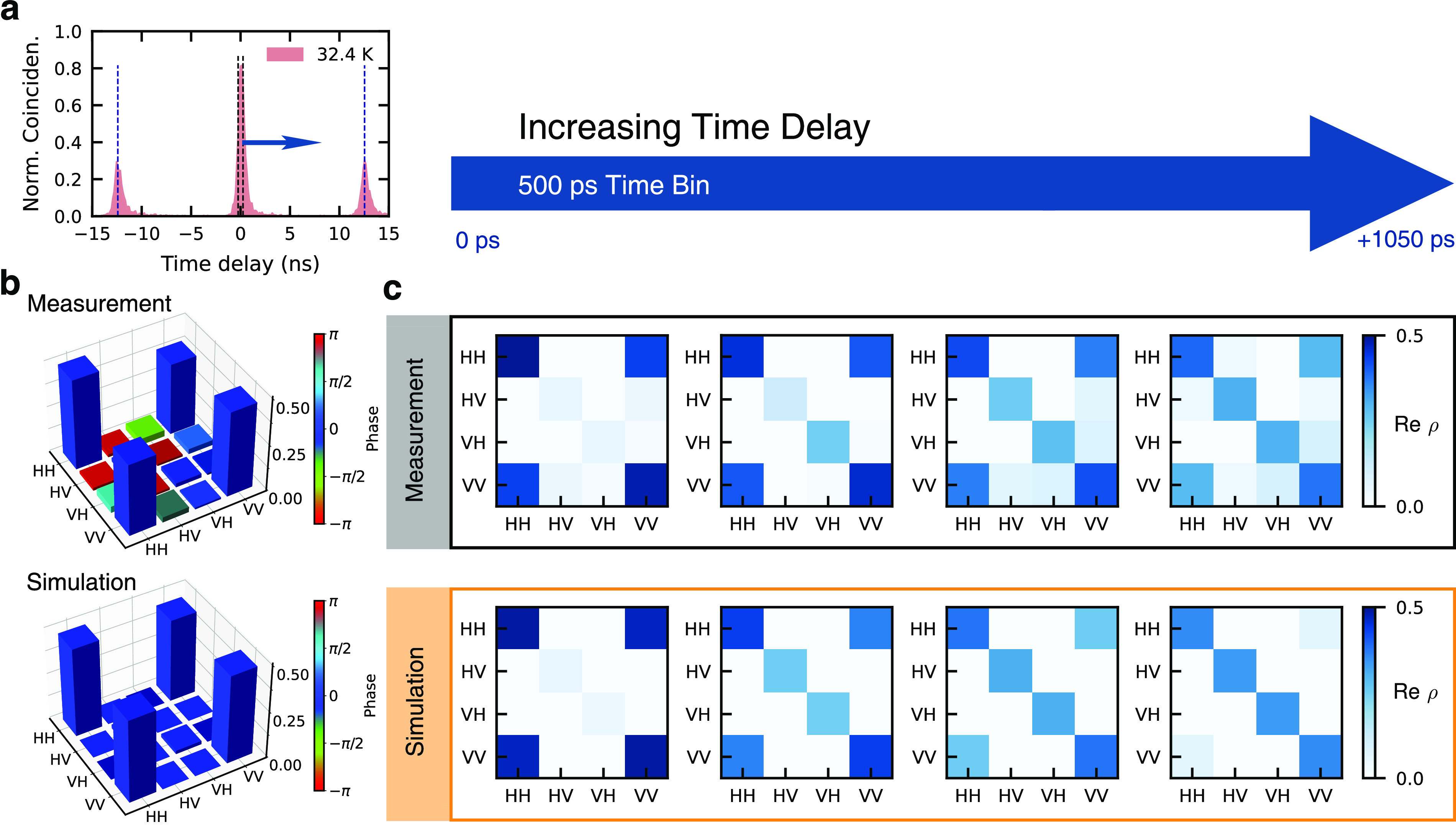
Comparison of measured and calculated two-photon density matrices at a temperature of 32.4 K for a time bin of 500 ps and different bin centers. (a) Example of XX-X coincidence histogram with a broadening on the right side of each peak stemming from slow X decay. Black dashed lines show the time bin used to calculate the density matrices shown in (b). The blue arrow shows the direction in which the time bin window is moved for time filtering beginning at zero time delay with the bin shown in (a). (c) 2D representations of the real parts of the density matrices with increasing time delays in equidistant steps, as obtained from the experiment (gray box) and theory (orange box). The leftmost matrices correspond to the time bin shown in (a) and used for (b).

In summary, we have investigated the effect of rising operation temperature on the quality of the polarization entanglement of photon pairs generated by the biexciton–exciton decay cascade in a single GaAs QD tuned to have negligible excitonic fine-structure splitting. By performing full-state tomography including time-filtering and lifetime measurements under resonant optical excitation as well as dedicated calculations, we ascribe both the entanglement degradation and the changes in decay dynamics to thermal cycling among the desired |*XX*⟩ and |*X*_*H*/*V*_⟩ states and “undesired” hot and dark states, which are connected to the former by phonon-assisted transitions leading to spin scattering and decoherence. In turn, the spin-agnostic character of the transitions is traced back to the high valence-band mixing in the excited states of the employed QDs.

From the achieved understanding one could envision that an increased energy splitting *ΔE* can substantially extend the plateau of high concurrence at lower temperatures up to 40 K, reachable with available Stirling coolers.^23,41^ Since the excited states will be less populated for larger energy splittings, the impact of thermal cycling will be reduced. An increasing *ΔE* is also expected to lead to a reduction of hole mixing in the excited states benefiting the preservation of high entanglement. Consequently, we anticipate that QDs capable of generating highly entangled photons with relaxed operation-temperature requirements can be obtained by slightly reducing the QD size. For GaAs QDs, this can be simply achieved by reducing the amount of GaAs filling.^42^ Recent work^50^ on (presumably strongly confining) InGaAs QDs shows indeed the persistence of high levels of entanglement beyond 90 K. However, along with a change in QD size one must consider also pure dephasing^43^ due to the deformation potential coupling to longitudinal acoustic (LA) phonons.^44−46^ This mechanism has been shown to reduce the concurrence by enhancing off-resonant single-photon transitions and decoherence.^47^ Although the effects of pure dephasing seem to be insignificant for the QD studied in this work, they become more relevant with decreasing QD size as the coupling to LA phonons becomes more effective.^48,49^. In conclusion, finding the optimal structural properties of QDs capable of emitting highly entangled photons at elevated temperatures will need further understanding of various effects and their impact.

## Data Availability

The data of this study is available from the corresponding author upon request.

## Supporting Information Available

Additional experimental details, materials and methods, and new references (PDF)
