# Supplementary material for: Beyond the Four-Level Model: Dark and Hot States in Quantum Dots Degrade Photonic Entanglement
Source: Nano Lett. 2023 Feb 6;23(4):1409–15. doi: 10.1021/acs.nanolett.2c04734 (PMC9951244; doi:10.1021/acs.nanolett.2c04734)
Supplement: Supplementary file 1 — nl2c04734_si_001.pdf [file nl2c04734_si_001.pdf]

Supporting Information: Beyond the four-level model: Dark and hot states in quantum dots degrade photonic entanglement

Barbara Ursula Lehner,*,†,‡ Tim Seidelmann,¶ Gabriel Undeutsch,† Christian Schimpf,† Santanu Manna,† Michał Gawełczyk,§ Saimon Filipe Covre da Silva,† Xueyong Yuan,‖ Sandra Stroj,⊥ Doris E. Reiter,# Vollrath Martin Axt,¶ and Armando Rastelli*,†

†*Institute of Semiconductor and Solid State Physics, Johannes Kepler University, Linz, 4040, Austria*

‡*Secure and Correct Systems Lab, Linz Institute of Technology, 4040 Linz, Austria*

¶*Theoretische Physik III, Universität Bayreuth, 95440 Bayreuth, Germany*

§*Department of Theoretical Physics, Faculty of Fundamental Problems of Technology, Wrocław University of Science and Technology, 50-370 Wrocław, Poland*

‖*School of Physics, Southeast University, Nanjing 211189, China*

⊥*Forschungszentrum Mikrotechnik, FH Vorarlberg, 6850 Dornbirn, Austria*

#*Condensed Matter Theory, TU Dortmund, 44221 Dortmund, Germany*

E-mail: barbara.lehner@jku.at; armando.rastelli@jku.at

Complementary Data

Sample structure

For the study of temperature dependent entanglement, GaAs quantum dots (QDs) grown by the local droplet etching method are used. The structure of the sample is shown in Figure 1. An (001)-oriented GaAs substrate is overgrown by a 475-nm-thick buffer layer, followed by nine pairs of alternating $Al_{0.95}Ga_{0.05}As/Al_{0.20}Ga_{0.80}As$ layers with a thickness of 69 nm/60 nm respectively, forming a distributed Bragg reflector. Next, a single 65 nm thick layer of $Al_{0.33}Ga_{0.67}As$ that hosts the droplet etched nanoholes is grown. By filling the nanoholes via deposition of 1.1 nm of GaAs followed by a 30 s annealing step the QDs are obtained, which are overgrown by another layer of $Al_{0.33}Ga_{0.67}As$ with 59 nm and a layer of 60 nm $Al_{0.2}Ga_{0.8}As$. The cavity is completed by two pairs of $Al_{0.95}Ga_{0.05}As/Al_{0.20}Ga_{0.80}As$ layers and a 4 nm GaAs cap. In the following, the sample is lapped down to ~ 30 µm and

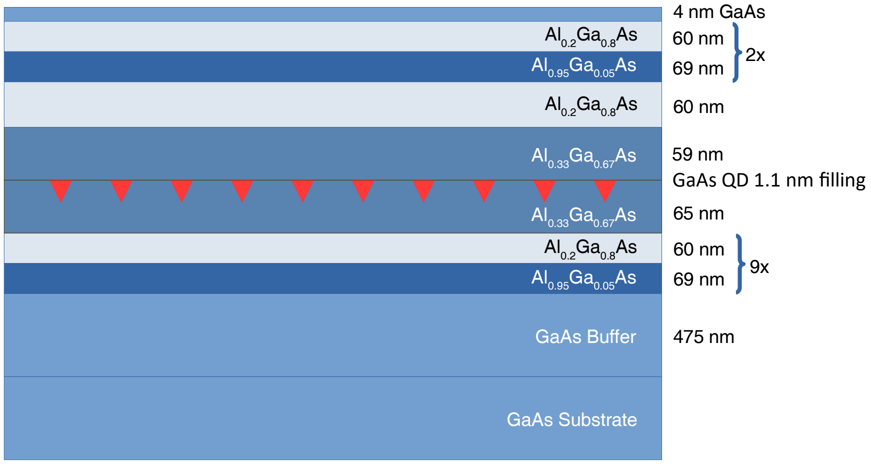

Figure 1: Structure of the sample used for temperature dependent entanglement measurements.

bonded with SU-8 resist to a 200 µm thick $[Pb(Mg_{1/3}Nb_{2/3})O_3]_{0.72} - [PbTiO_3]_{0.28}$ (PMN-PT) piezo featuring six micro-machined legs in order to apply three independent quasi-uniaxial stresses through Cr/Au contacts.[1,2] Figure 2 shows the above-band spectra of a representative QD at low temperature and the tunability of the fine-structure splitting (FSS) by applying

voltages to the so-called six leg device. The device is mounted and contacted in a He bath cryostat ensuring stable temperatures during measurement. For the optical excitation and collection of the PL signal an aspheric lens was used. For the temperature-dependent measurements presented in the main paper, two-photon resonant excitation (TPE) was used. The FSS was then determined by performing linear-polarization-resolved PL measurements of the exciton (X) and the biexciton (XX) lines, with an uncertainty of 0.2 µeV. At each temperature the FSS was found to change by 0.5 µeV − 1 µeV, most probably because of strain changes due to variations in the piezoelectric coefficients of the strain actuator[3] and the mismatch in thermal expansion coefficients of the piezoelectric and semiconductor material.

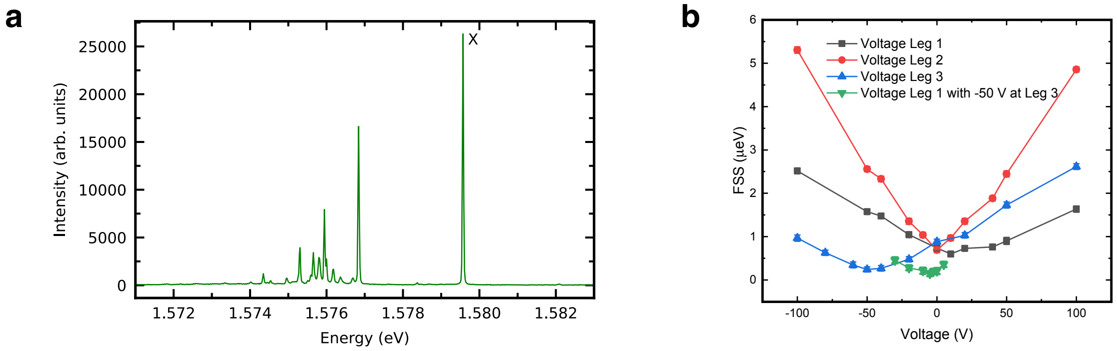

Figure 2: (a) Representative spectrum under above-band excitation at around 5 K of a GaAs QD of the sample in Figure 1. The exciton line is marked with "X". (b) The excellent tunability of fine-structure splitting (FSS) of about 5 µeV within ±100 V in this sample is shown for each of the three leg pairs with applied voltages (grey, red, and blue). By combining different strain axes (green line) the FSS can be tuned to the resolution limit.

Auto-correlation measurements

Figure 3 shows the values of the second-order auto-correlation function at zero time-delay [$g^{(2)}(0)$] for biexciton (XX) and exciton (X) at temperatures up to 64.4 K.

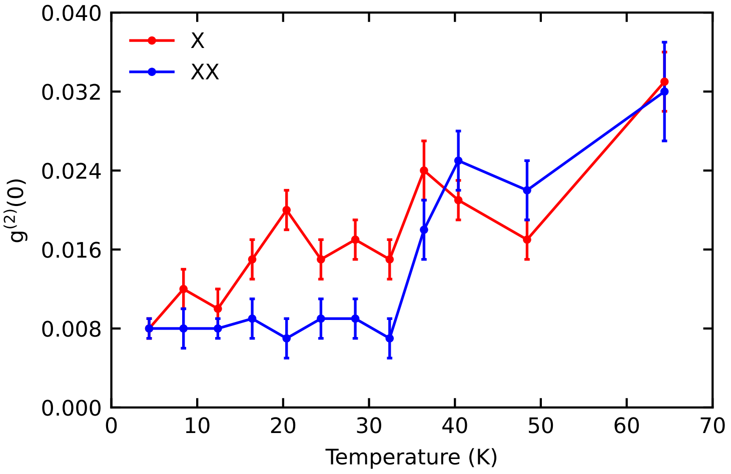

Figure 3: $g^{(2)}(0)$ values for biexciton XX and exciton X for temperatures up to 64.4 K for a time bin of $\Delta t = 2$ ns.

Decay dynamics

Lifetime of biexciton and exciton at 4.4 K

The extraction of biexciton XX and exciton X lifetime at 4.4 K is done by performing a convolutional fit (see Figure 4) that takes the instrument response function (IRF) into account. The biexciton decay was fitted with a monoexponential function, the exciton decay with a double exponential, resulting in a lifetime of XX of 129(3) ps and in an X lifetime of 231(4) ps.

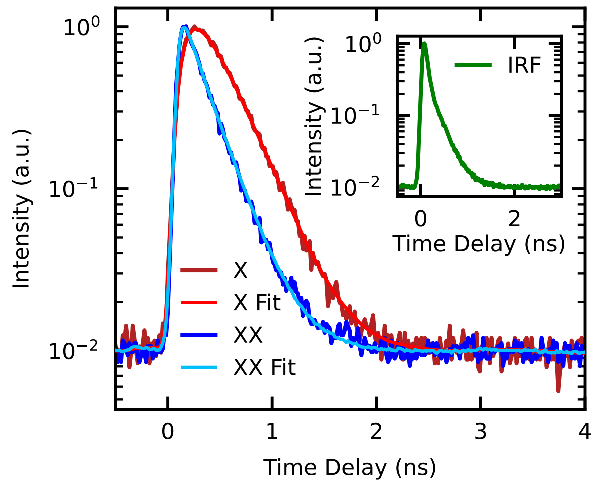

Figure 4: Evaluation of decay traces of biexciton XX and exciton X at 4.4 K by performing a convolutional fit with the instrument response function IRF (inset).

Rate equations

For the presented model [see Figure 2(b) in the main paper], the occupation of the states can be calculated by setting up the rate equation system given by:

$$\begin{pmatrix} \dot{n}_{XX^*} \\ \dot{n}_{XX} \\ \dot{n}_{X^*} \\ \dot{n}_{X} \\ \dot{n}_{X_D} \end{pmatrix} = \begin{pmatrix} -\gamma_{XX_1^*} - \gamma_{XX_2^*} - \gamma_{PH2} - \gamma_{XX_D^*} & 4\gamma^*_{PH2} & 0 & 0 & 0 \\ \gamma_{PH2} & -\gamma_{XX} - 4\gamma^*_{PH2} & 0 & 0 & 0 \\ \gamma_{XX_2^*} & 0 & -\gamma_{X^*} - \gamma_{PH} - 2\gamma_{PH_D} & 4\gamma^*_{PH} & 4\gamma^*_{PH_D} \\ \gamma_{XX_1^*} & \gamma_{XX} & \gamma_{PH} & -\gamma_X - 4\gamma^*_{PH} & 0 \\ \gamma_{XX_D^*} & 0 & 2\gamma_{PH_D} & 0 & -4\gamma^*_{PH_D} \end{pmatrix} \begin{pmatrix} n_{XX^*} \\ n_{XX} \\ n_{X^*} \\ n_{X} \\ n_{X_D} \end{pmatrix} \quad (1)$$

where n is the probability of occupation of the given states. The dot indicates the time derivative. The rates γ correspond to the rates given in the main text. Due to resonant two-photon excitation the system is prepared in the $|XX\rangle$ state, which is defined by the

following initial conditions:

$$\begin{aligned}
n_{XX^*}[t=0] &= 0, \\
n_{XX}[t=0] &= 1, \\
n_{X^*}[t=0] &= 0, \\
n_{X}[t=0] &= 0, \\
n_{X_D}[t=0] &= 0.
\end{aligned} \tag{2}$$

By solving the rate equations and using the rates in Table 1 of the main text, one obtains the decay traces shown in Figure 2(a) of the main paper. The slope and the slow decay of X is dominated by the slow phonon-assisted rates γ^*_{PH} and γ^*_{PH2} of the system *and* the state multiplicity of 4 for $|X^*\rangle$ and $|XX^*\rangle$ as long as γ_{X^*} is slower than γ_X decay.

Having a closer look to the XX decay (Figure 2(a) of the main paper), we see that in the simulation no crossing of high and low temperature decay curves is visible compared to the measurement. We attribute this to the fact that we were not able to explicitly determine the energy distance $|XX^*\rangle - |XX\rangle$. Therefore, the same energy separation (3.7 meV) as for the $|X^*\rangle$-$|X\rangle$ splitting was assumed. By using a smaller $|XX^*\rangle - |XX\rangle$ energy of, e.g., 1.4 meV the presented model can reproduce the crossing of the X decay curves as shown in Figure 5. However, the smaller energy separation is also affecting the X decay resulting in a much slower decay for lower temperatures than observed in the measurement. On the contrary, the predicted X decay as it is shown in Figure 2(a) of the main paper is faster at higher temperatures than in the measurement, which can be attributed to further excited states appearing at high temperatures (X^{**} and X^{***}) that were not considered in the model (see section “Further excited states”).

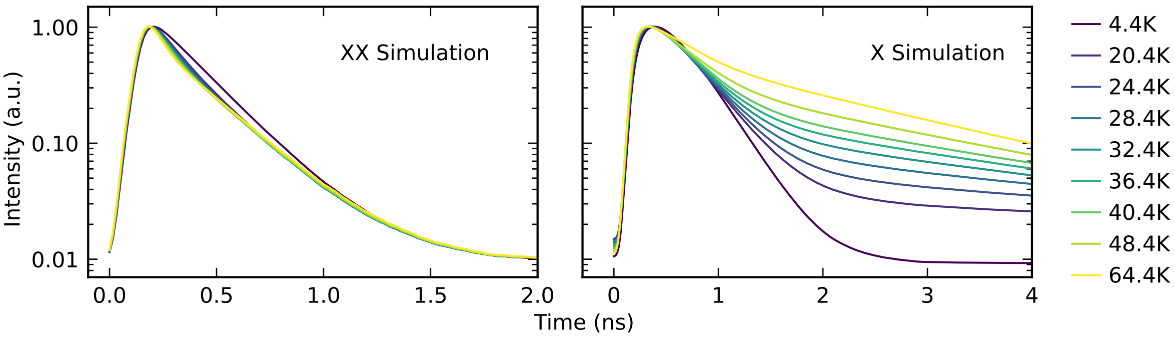

Figure 5: Simulated decay dynamics of XX and X decay with a modified $|XX^*\rangle - |XX\rangle$ energy distance of 1.4 meV. Note the different time scales for XX and X. For the XX decay a crossing of high and low temperature decays is visible at around 0.6 ns.

Populating the excited exciton $\mathbf{X}^*$

The $|X^*\rangle$ is an excited state of the neutral exciton. In a single particle picture, it consists of an s-shell electron and a p-shell hole, which produce a spectral line labeled X^* upon radiative recombination. By preparing the system in the $|X^*\rangle$ state via resonant excitation one can measure the exciton decay and compare it with the calculated occupation from the rate equations with the parameters given in the main paper. For this measurement a charge-tunable diode sample with comparable GaAs QDs was used. Figure 6 shows the simulated decay curve of X convolved with a measured IRF in red at 5 K by using the same rates as given in the main paper. The measured exciton decay is shown in dark red. A good match between model and experiment is found, considering that a different QD was measured here compared to the one used to develop the rate equation model. Further, measurements with populated $|X^*\rangle$ show equal intensities of X_V and X_H (vertically and horizontally polarized excitonic emission) regardless of laser polarization, confirming the loss of polarization information, which is linked to the spin properties of the involved states.

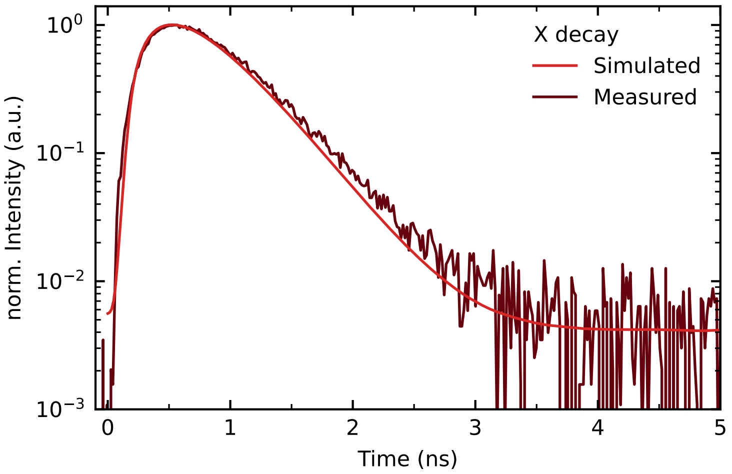

Figure 6: Exciton decay measured by preparing the system initially in the excited $|X^*\rangle$ state with a laser pulse tuned to the X^* line. The measured decay on a comparable sample is shown in dark red, the simulated decay is shown in light red. The simulation considers the convolution with the instrument response function.

Concurrence

In order to determine the photonic density matrix and the degree of entanglement between X and XX, a tomographically complete set of 16 cross-correlation measurements corresponding to different polarization configurations according to Ref. 4 was performed. The analysis of these measurements was accomplished by comparing the coincidences in a certain time window (time bin) with the average side peak areas and using a maximum likelihood estimation.[4] A time bin of 2 ns was used in the concurrence evaluation and simulation of Figure 1 in the main paper, leading to the inclusion of almost all detected events. Whereas a time bin of different duration does not produce appreciably different results at low temperatures (because we have tuned the FSS to negligible values and thermal cycling events are suppressed) the tail in the coincidence histograms at higher temperatures is associated to spin scattering and decoherence. This means that if the exciton photon is emitted about a nanosecond later than the biexciton photon at elevated temperatures, the system was most probably in an excited state or in a dark exciton state before returning to the bright exciton state, resulting in the loss of polarization and coherence. Hence, by evaluating the coincidence measurements

with a sufficiently long time bin, more of these events are included, resulting in a lower concurrence for longer time bins, as shown in Figure 7. With a time bin of 8 ns practically all delayed coincidences at a temperature of 64.4 K are included in the concurrence evaluation. However, it is of high importance that measurement evaluation and simulation (see section "Photonic 2-qubit density matrix and concurrence") consider the same time bin. Figure 7 shows good agreement between measurement and simulation for different time bins up to 40 K. For temperatures > 40 K the mismatch between theory and experiment is larger at the 8 ns time bin, due to fact that the theory does not take further excited states into account. However, the main influence on the concurrence with the considered rate model of this work comes from the ratios γ^*_{PH}/γ_X and $\Delta E/T$.

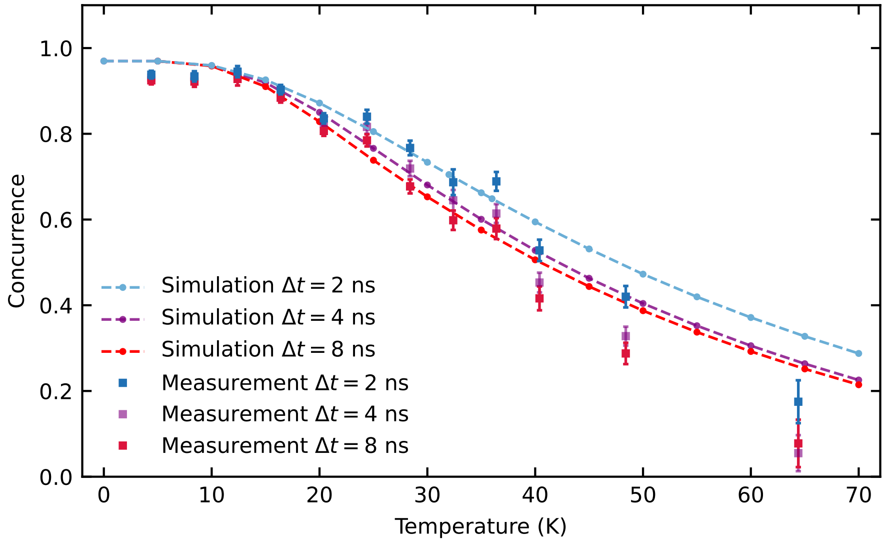

Figure 7: Concurrence obtained by evaluating the measurement (dots) with different time bin widths (2 ns, 4 ns, 8 ns), which was also used in the simulations (dashed lines).

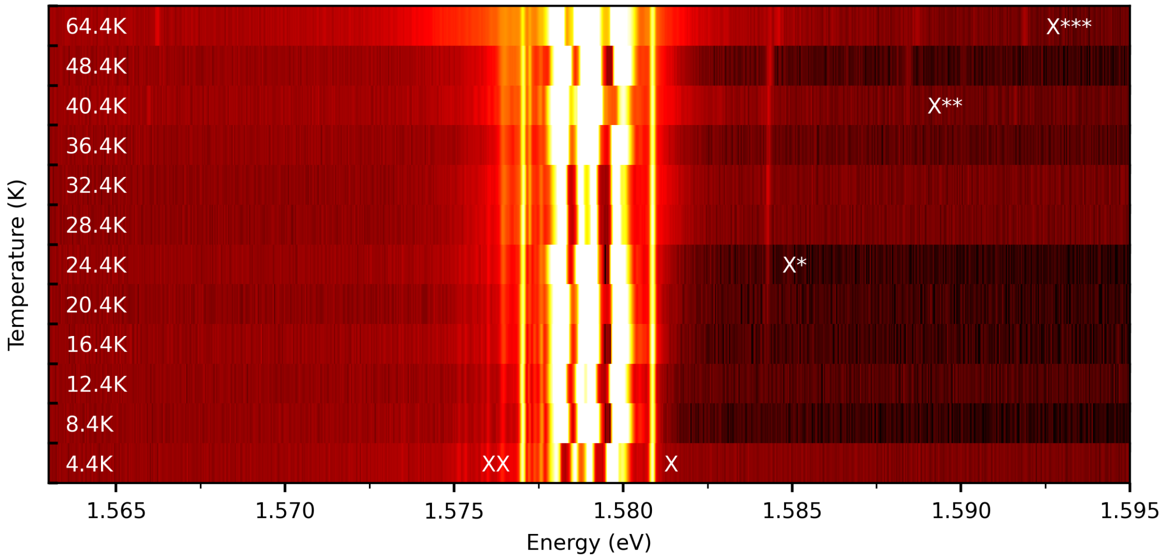

Figure 8: Color coded PL spectra of the same QD treated in the main text at different temperatures up to 64.4 K. For enhancing the signal to noise ratio, at each temperature the spectra of a polarization-resolved measurement were averaged. Two-photon-excitation is used and residual laser stray light is seen between the XX and X lines. The spectral red shift due to temperature is subtracted here.

Further excited states

Results of the main paper show that simulations start to deviate from the measurements at higher temperatures. We attribute these deviations to the exclusion of further excited states in the theoretical model. Figure 8 shows that at 40.4 K and 64.4 K transitions from additional excited states (X^{**} and X^{***}) start to appear in the micro-photoluminescence spectra. We can extract from the measurements that X^* lies 3.7 meV above the X, the X^{**} 4.1 meV above X^*, and X^{***} 3.5 meV above X^{**}. Further, we make use of the intensities extracted from the PL spectra shown in Figure 8 to estimate the decay rate γ_{X^*}. We use the relation

$$\frac{\gamma_{X^*} \int n_{X^*} \, \mathrm{d}t}{\gamma_X \int n_X \, \mathrm{d}t} \propto \frac{I_{X^*}}{I_X}, \tag{3}$$

where $n_{X^*}(n_X)$ is the occupancy of the $|X^*\rangle(|X\rangle)$ state and $I_{X^*}(I_X)$ the extracted PL intensities of X^*(X) lines. From the intensity ratio extracted out of the PL data we estimate

the rate $\gamma_{X^*} \approx \frac{1}{10\,\mathrm{ns}}$. This value is also in good agreement with the theory results (see below), where we get $\sim$ 8 ns and $\sim$ 15 ns lifetimes for the two bright X* states, which gives an average recombination rate of $(1/8 + 1/15)/2$ ns^{-1} $\simeq$ 0.1 ns^{-1}. Further, we compare biexciton and exciton intensity ratio from theory and experiment. Figure 9 shows that also the simulated and measured XX/X ratios are of the same order and follow the trend of the theory, indicating that the XX intensity becomes lower with higher temperatures due to the population of the $|XX^*\rangle$ hot states. This confirms that our kinetic model (see section: "Transition processes and equation of motion"), equipped with the adopted energy splittings and the calculated rates of phonon-assisted transitions (see section: "Phonon-assisted relaxation"), reflects well the thermal changes of the state occupation and, thus, the intensity of the radiative transitions.

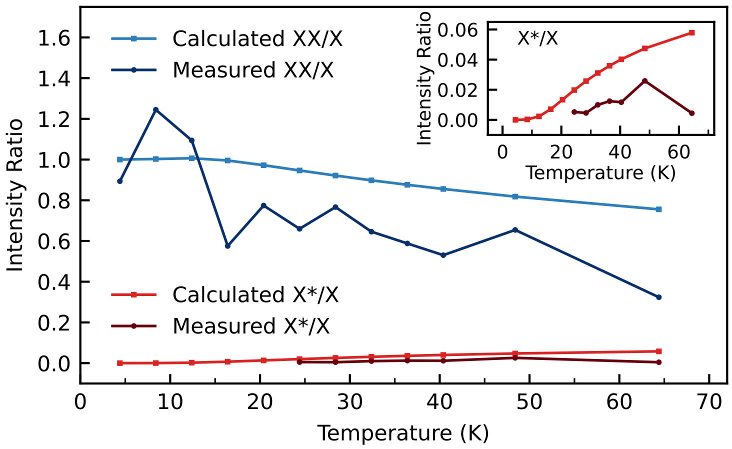

Figure 9: Calculated and measured intensity ratios.

Theoretical Model and Simulations

Modeling of quantum dot electronic structure

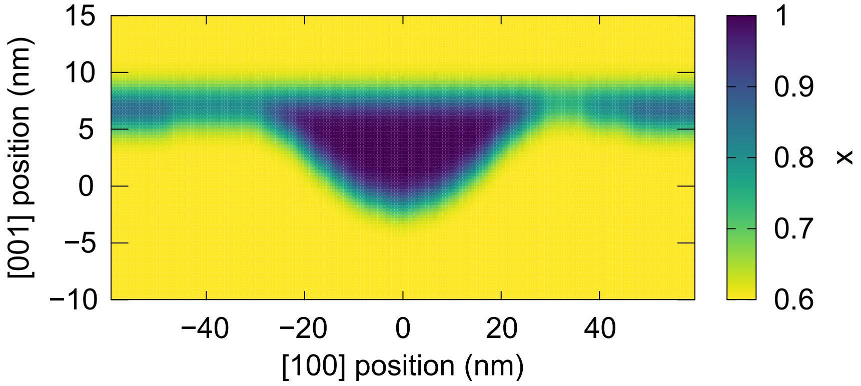

Figure 10: A Cross-section of the modeled QD material composition with bottom interface based on the AFM image of a representative nanohole. The color indicates the local x value in $Al_{1-x}Ga_xAs$.

We model the considered QDs based on available morphological data. We represent the simulated QDs on an axis-wise uniform numerical grid of the local material composition values. The shape of the lower AlGaAs-GaAs interface in the simulation comes directly from atomic force microscopy (AFM) measurements of nanoholes left after droplet etching. We assume that the top GaAs surface formed is flat. We then apply Gaussian averaging with $\sigma = 1.2$ nm of the three-dimensional material composition profile to simulate the interdiffusion of atoms at interfaces well described by normal diffusion. The resultant profile of material composition (given by the value of x in $Al_{1-x}Ga_xAs$) is shown in Figure 10 of the Supplementary Material. Although the lattice mismatch of the considered materials is minimal, we still calculate possible structural strain within the theory of continuous elasticity. Then we also calculate the piezoelectric field, resulting from the shear strain generated at the interface, with terms in piezoelectric polarization up to second order in strain-tensor elements.

Carrier eigenstates

For such a simulated structure, we calculate the eigenstates of electrons and holes using the implementation of the multiband $\boldsymbol{k} \cdot \boldsymbol{p}$ method within the envelope-function approximation[5] described in Ref. 6. It includes effects caused by the spin-orbit interaction, structural strain, and piezoelectric field. The explicit form of the Hamiltonian may be found in Ref. 7, while material parameters used in calculations are given in Ref. 8 and references therein. Via numerical diagonalization, we obtain the carrier eigenvalues and eigenvectors $\boldsymbol{\psi}$ in the form of eight-component pseudospinors. Their components are the discretized envelope functions within each of the eight considered subbands (conduction, heavy-hole, light-hole and spin-orbit split off bands). We apply the time-reversal operation to the valence-band electron eigenstates to obtain hole states.

By calculating a number of the lowest-energy states of both types of carriers, we determine the density of their energy ladders. We find the splittings between the ground and first excited states to be 2.93 meV and 17.2 meV for holes and electrons, respectively. We obtain up to $\sim 20\%$ variation of these splittings within possible inaccuracies in mapping the exact dot morphology. We also find that weak strain and hence minimal nominal splitting of heavy- and light-hole subbands lead to substantial hole subband mixing. While the hole ground state is of a predominantly heavy-hole character with only a few percent ($\sim 6\%$) light-hole admixture, already the first excited state is almost fully mixed, with a total light hole admixture of about 38 % (approx. contributions: 10.6 % $|3/2\rangle$, 24.8 % $|+1/2\rangle$, 13.4 % $|-1/2\rangle$, 50.9 % $|-3/2\rangle$ for one of the states with the other being its spin-flipped counterpart). This leads to the mismatch of spin bases between the ground and excited hole states with pronounced consequences for the recombination and phonon-assisted relaxation.

Excitonic states

We use a basis of up to 56 electron and 56 hole eigenstates to construct the configuration bases for carrier complexes. By diagonalizing Hamiltonians of Coulomb interaction and

phenomenological electron-hole exchange interaction (with fine-structure splittings based on the experimental data), we obtain the eigenstates of excitons and biexcitons.

For an accurate calculation, an infinite configuration basis would be needed. To obtain satisfactorily convergent results, we repeat all calculations for excitons and biexcitons for an increasing number of configurations. We then extrapolate the asymptotic behavior of the results with a smooth function.

In such a way, we obtain the exciton binding energy equal to 19.4 meV. The much denser ladder of hole states than electron ones leads to the fact that a large initial part of the exciton spectrum consists of orbital states composed mainly of the electron in the ground state bound with the hole in its successive excited states. The energy distance to the first excited shell is ~ 3 meV, which is reasonably in line with the 3.7 meV observed in the experiment. Each orbital state corresponds to four eigenstates with different spin configurations. We also find the biexciton binding energy to be $E_{XX} - 2E_X \simeq -3.4$ meV, with relatively high uncertainty as we are able to use maximally 40 electron and 40 hole states for the XX calculation (the size of configuration space grows exponentially with the number of particles).

Next, we calculate the optical transition dipole moments[9] and the corresponding oscillator strengths (and state lifetimes)[10] within the dipole approximation to determine the optical properties of calculated eigenstates. In the case of the ground state, we are dealing with a standard pair of dark states and a pair of bright states that couple to light predominantly linearly polarized along the [110] and [1$\bar{1}$0] axes. The substantial light-heavy hole mixing within excited hole levels translates into pronounced mixing of heavy- and light-hole exciton states in the excited shells.

Nominally, the bright states of an exciton are evenly mixed (through the electron-hole exchange interaction) states that couple to circular polarizations. This results in eigenstates emitting linearly polarized light. Light-hole admixtures (those of opposite spin, while those with the spin aligned with the main heavy-hole contribution are dark) produce contributions of opposite circular polarization to those nominally circular contributions. For a regular

orbitally bright state, this leads to unequal oscillator strengths of the two states and modification of their polarization properties. Here, we deal with optical activity of states that should be nominally dark, as they involve an odd wave-function envelope for the electron and an even one for the hole. However, the light-hole admixture within the first excited hole state involved here has an s-type symmetry, which enables optical transitions. The calculated radiative rates for the bright ground states are $\gamma_{\mathrm{X}} \simeq 1/350\ \mathrm{ps}^{-1}$, while for the excited states we estimate the lifetimes to be ~ 8 ns and ~ 15 ns (uncertainty of extrapolation is higher here). For the sake of model simplicity, we take an average rate (harmonic mean of lifetimes) for these two equal $0.1\ \mathrm{ns}^{-1}$.

For the XX, we get $\gamma_{\mathrm{XX}} \simeq 1.74\gamma_{\mathrm{X}}$, which is very close to the ratio obtained experimentally. Optical transitions from the excited levels are strongly affected by light-hole admixtures. As mentioned, they result in mismatched spin configurations between the ground and excited states. Additionally, as underlined in the discussion of X^* transitions, these admixtures also allow for transitions between states with nominally mismatched envelope parity. As a result, we deal with recombination from XX^* to both X and X^* levels. For the $\mathrm{XX}^* \rightarrow \mathrm{X}^*$ one, we get the total rate of $\sim 1/0.5\ \mathrm{ns}^{-1}$, while for other, $\mathrm{XX}^* \rightarrow \mathrm{X}$, we were able to estimate the rate to be $\sim 1/20\ \mathrm{ns}^{-1}$. These values, however, are subject to greater uncertainty because, in the case of the biexciton, we were able to use the basis of maximally 32 electron and 32 hole states. Nevertheless, these calculations justify the values of XX^* recombination rates adopted in the kinetic model.

Phonon-assisted relaxation

We consider the interaction of carriers with bulk acoustic phonons in the long-wavelength limit. To this end, we take into account the deformation potential and piezoelectric couplings, as described explicitly in Ref. 11. Both of these couplings enter the Hamiltonian through a phonon-induced oscillating strain field. The coupling via the deformation potential is calculated by inserting the phonon strain field into the Bir-Pikus Hamiltonian,[12] while the

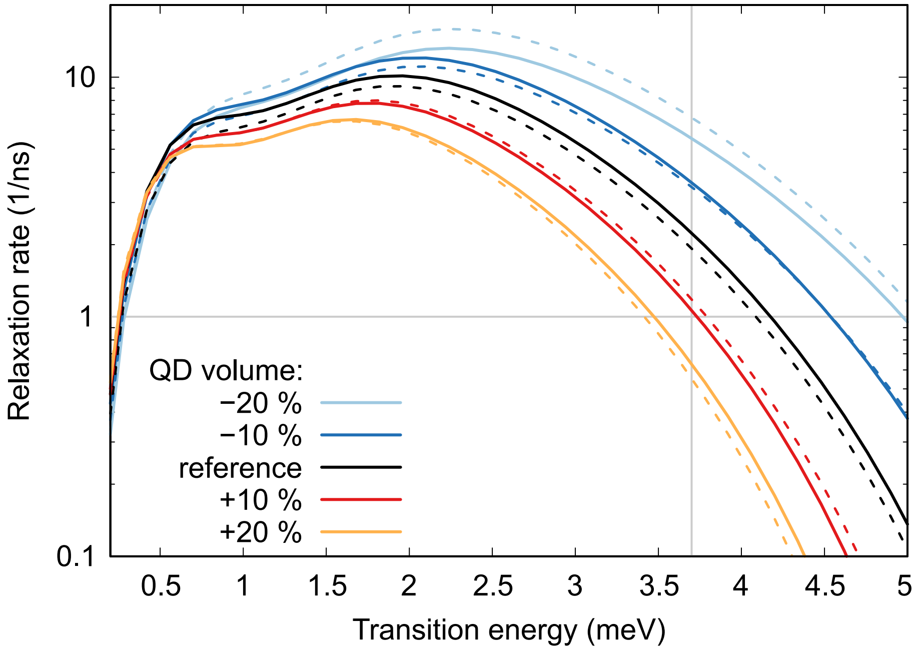

Figure 11: Phonon-assisted hole relaxation rates as a function of transition energy. The black lines show the rate for the reference QD model (solid and dashed for two different initial-final spin configurations), while other lines correspond to modified QD volume. The vertical pale line shows the X*-X transition energy determined experimentally, and the horizontal line shows the relaxation rate assumed in the kinetic model.

piezoelectric coupling is taken into account through the piezoelectric field induced by phonon-induced shear strain. We calculate the transition rates within the Fermi golden rule. For a transition between a pair of exciton states, the relaxation rate is obtained by summing all the contributions for pairs of configuration basis components that differ in the state of one of the particles (we consider only one-phonon processes). For this reason, the computation is highly resource and time consuming and we are limited here to the basis of maximally 24 electron and 24 hole states.

Directly from the excitonic calculation, we get the relaxation rate of $\gamma^0_{PH} \sim 4$ ns^{-1}. However, this value is not well converged due to insufficient configuration basis size achievable in our calculation, so the uncertainty is relatively high. Moreover, the calculated transition energy differs from the experimentally determined one. This discrepancy indicates a slight difference in geometry between the experimentally investigated QD and the model based on the specific nanohole. While the other results are less sensitive to minor changes in

dot morphology, the phonon relaxation rate strongly depends on the QD volume. This dependence is because the considered transition falls on the quickly vanishing high-energy tail of the phonon spectral density. To analyze this effect, in Figure 11 of the Supplementary Material we show the hole relaxation rate from the first excited state to the ground state as a function of the transition energy. Different line types correspond to different combinations of spin states. The analysis of the transition rate in a single-particle language is largely justified by the fact that the X state in 95 % consists of the e1h1 configuration, and the X* state in 94 % consists of e1h2 (where the numbers refer to the orbital states of the electron and hole, respectively). The black curve corresponds to the reference dot, while the red and blue curves with different saturation correspond to 10 % and 20 % smaller/larger QDs. We notice that the rate $\gamma^0_{PH} = 1$ ns^{-1} we assume in the kinetic model corresponds to a dot 10 % larger than the reference one, which is well within the uncertainty associated with modeling this type of nanostructure.

Most importantly, it should be noted that this relaxation is much slower than typically expected for excitons confined in quantum dots. This feature is due to the large volume of the dots considered here. Additionally, the calculations allowed us to establish an even more important fact. Namely, all pairs of X* and X states (different combinations of spin configurations) have comparable transition rates (differences within 40% margin according to the excitonic calculation). It is caused by the above-described mismatch of the spin configurations in the ground and excited exciton states, which results from the mixing of valence subbands in the excited states of the hole. We exploit this fact in constructing the kinetic model by assuming all transitions have the same rate.

Level structure and optical excitation

For our simulations, we extend the QD model presented in Ref. 13 by introducing further states and a more detailed temperature dependence for the phonon-induced transition rates.

In addition to the standard biexciton-exciton-cascade, comprising the biexciton $|XX\rangle$,

two bright exciton states $|X_{H/V}\rangle$ that couple to horizontally (H) and vertically (V) polarized light, and the ground state $|G\rangle$, the model accounts for excited biexciton $|XX^*\rangle$ and exciton states $|X^*\rangle$ as well as dark excitons $|X_D\rangle$. Furthermore, the biexciton is excited by a two-photon resonant laser pulse with horizontal polarization. In a frame co-rotating with the (central) laser frequency, the Hamiltonian describing the QD under TPE is given by

$$\begin{aligned}\hat{H}_{\text{sys}} =& \frac{E_B}{2}\left(|X_H\rangle\langle X_H| + |X_V\rangle\langle X_V|\right) + \left(\frac{E_B}{2} + \Delta E\right)|X^*\rangle\langle X^*| + \Delta E\,|XX^*\rangle\langle XX^*| \\ &+ \left(\frac{E_B}{2} - \delta_{BD}\right)|X_D\rangle\langle X_D| - \frac{\hbar}{2}\Omega(t)\left(\hat{\sigma}_H + \hat{\sigma}_H^\dagger\right)\end{aligned} \tag{4}$$

where the energy of the ground state is used as the zero of the energy scale. Note that, in this rotating frame, the energy of the biexciton is also zero. Here, E_B is the biexciton binding energy, ΔE denotes the energy difference between the excited and ground exciton states, and δ_{BD} is the bright-dark splitting. The parameter values $E_B = 4\,\text{meV}$, $\Delta E = 3.7\,\text{meV}$, and $\delta_{BD} = 110\,\mu\text{eV}$ [14] are taken from measurements. For simplicity, the energy difference between excited and ground biexciton is also set to ΔE. Furthermore, in accordance with the experiment, the fine-structure splitting between the two bright excitons is zero.

In order to describe the experimentally determined intensity profile of the laser pulse, the (real) pulse envelope

$$\Omega(t) = \frac{\Theta}{\sqrt{2\pi}\,\sigma}\left\{e^{-\frac{1}{2}\left(\frac{t-t_L}{\sigma}\right)^2} + R\left[e^{-\frac{1}{2}\left(\frac{t-t_L+t_S}{\sigma}\right)^2} + e^{-\frac{1}{2}\left(\frac{t-t_L-t_S}{\sigma}\right)^2}\right]\right\} \tag{5}$$

is approximated as three subsequent Gaussian peaks, cf., Figure 12. Here, t_L denotes the time of the pulse maximum and Θ is the pulse area of the central peak. The corresponding intensity $I(t) \propto \Omega^2(t)$ fits well to the pulse form in the experiment for the parameter set: $\sigma = 3.48\,\text{ps}$, $R = 0.37$, and $t_S = 12.4\,\text{ps}$. The optimal pulse area $\Theta \approx 4.4\pi$ is determined numerically by optimizing for the maximum biexciton occupation during the system dynamics. Since a horizontal laser polarization is assumed the laser pulse couples to the QD transitions described by the operator $\hat{\sigma}_H = |G\rangle\langle X_H| + |X_H\rangle\langle XX|$.

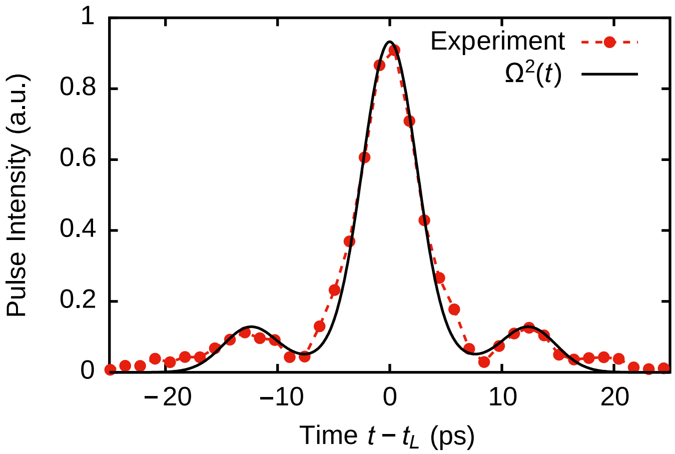

Figure 12: Pulse intensity determined in the experiment (red dots) and $I(t) \propto \Omega^2(t)$ according to Equation (5) for $\sigma = 3.48\,\mathrm{ps}$, $R = 0.37$, and $t_S = 12.4\,\mathrm{ps}$.

Transition processes and equation of motion

Radiative decay processes and temperature-dependent, phonon-induced transitions between the QD states are incorporated into the model by Lindblad operators[15]

$$\mathcal{L}(\hat{O}, \Gamma)\,\hat{\rho} = \frac{\Gamma}{2}\left(2\,\hat{O}\hat{\rho}\hat{O}^\dagger - \hat{O}^\dagger\hat{O}\hat{\rho} - \hat{\rho}\hat{O}^\dagger\hat{O}\right), \tag{6}$$

that act on the statistical operator $\hat{\rho}$ of the QD. Here, the operator $\hat{O}$ is the QD operator associated with the relevant processes that occurs with rate Γ.

The QD model presented in Figure 2(b) of the Main Text with all its states and transition

processes is described by the Liouville-von-Neumann equation

$$\begin{aligned}\frac{\mathrm{d}}{\mathrm{d}t}\hat{\rho} =&\mathcal{L}_{\mathrm{sys}}\,\hat{\rho} := -\frac{i}{\hbar}\left[\hat{H}_{\mathrm{sys}},\hat{\rho}\right] + \sum_{\ell=H,V}\left\{\mathcal{L}(\left|G\right\rangle\left\langle X_\ell\right|,\gamma_X) + \mathcal{L}(\left|X_\ell\right\rangle\left\langle XX\right|,\frac{\gamma_{XX}}{2})\right\}\hat{\rho}\\
&+\sum_{\ell=H,V}\mathcal{L}(\left|X_\ell\right\rangle\left\langle XX^*\right|,\gamma_{XX_1^*})\,\hat{\rho} + \mathcal{L}(\left|X^*\right\rangle\left\langle XX^*\right|,\gamma_{XX_2^*})\,\hat{\rho} + \mathcal{L}(\left|X_D\right\rangle\left\langle XX^*\right|,\gamma_{XX_D^*})\,\hat{\rho}\\
&+\mathcal{L}(\left|G\right\rangle\left\langle X^*\right|,\gamma_{X^*})\,\hat{\rho} + \sum_{\ell=H,V}\left\{\mathcal{L}(\left|X_\ell\right\rangle\left\langle X^*\right|,\gamma_{PH}(T)) + \mathcal{L}(\left|X^*\right\rangle\left\langle X_\ell\right|,4\gamma^*_{PH}(T))\right\}\hat{\rho}\\
&+\mathcal{L}(\left|XX\right\rangle\left\langle XX^*\right|,\gamma_{PH2}(T))\,\hat{\rho} + \mathcal{L}(\left|XX^*\right\rangle\left\langle XX\right|,4\gamma^*_{PH2}(T))\,\hat{\rho}\\
&+\mathcal{L}(\left|X_D\right\rangle\left\langle X^*\right|,2\gamma_{PH_D}(T))\,\hat{\rho} + \mathcal{L}(\left|X^*\right\rangle\left\langle X_D\right|,4\gamma^*_{PH_D}(T))\,\hat{\rho}\end{aligned} \tag{7}$$

which governs the dynamics of the statistical operator $\hat{\rho}$ of the QD. Here, $[\hat{A},\hat{B}]$ denotes the commutator of two operators $\hat{A}$ and $\hat{B}$. γ_X (γ_{XX}) is the rate associated with the radiative decay of an exciton (the biexciton) that leads to the emission of an exciton (biexciton) photon which is then detected in the coincidence measurement. The remaining radiative decay rates $\gamma_{XX_1^*}$, $\gamma_{XX_2^*}$, $\gamma_{XX_D^*}$, and γ_{X^*} correspond to transitions involving excited states, where the emitted photons do not appear in the coincedences.

Besides the radiative decay of various QD states, the model also accounts for transitions between states due to phonon emission and absorption processes. The temperature dependence of the corresponding rates

$$\gamma_{PH}(T) = [1+n(\Delta E,T)]\,\gamma^0_{PH};\quad \gamma^*_{PH}(T) = n(\Delta E,T)\,\gamma^0_{PH} \tag{8a}$$

$$\gamma_{PH2}(T) = [1+n(\Delta E,T)]\,\gamma^0_{PH};\quad \gamma^*_{PH2}(T) = n(\Delta E,T)\,\gamma^0_{PH} \tag{8b}$$

$$\gamma_{PH_D}(T) = [1+n(\Delta E+\delta_{BD},T)]\,\gamma^0_{PH};\quad \gamma^*_{PH_D}(T) = n(\Delta E+\delta_{BD},T)\,\gamma^0_{PH} \tag{8c}$$

is determined by the expected number of phonons with energy E

$$n(E,T) = \left(\exp\left[\frac{E}{k_B T}\right] - 1\right)^{-1} \tag{9}$$

excited at temperature T according to the Bose-Einstein statistics. For simplicity, we assume the rate γ^0_{PH} to be the same for all three phonon-induced transitions. Note that the prefactor 4 (2) in Equation (7) accounts for the fact that we assume four (two) energetically degenerate excited exciton and biexciton (dark exciton) states that are represented by one effective state $|X^*\rangle$ and $|XX^*\rangle$ ($|X_D\rangle$), respectively.

Equation (7) can be numerically solved based on its formal solution

$$\hat{\rho}(t) = \mathcal{P}_{0\to t}\left[\hat{\rho}(0)\right] := \hat{T}\exp\left[\int_0^t \mathrm{d}t' \mathcal{L}_{\mathrm{sys}}\right]\hat{\rho}(0) \tag{10}$$

where the formal propagator $\mathcal{P}_{0\to t}$ is introduced, and $\hat{T}$ denotes the time-ordering operator. For all simulations, we assume the QD to be initially in its ground state $|G\rangle$ before it is excited by the TPE pulse.

The parameter values for the different rates that are employed in the simulations are listed in Table 1 of the Main Text. Note that the rate γ^0_{PH} is the only remaining fit parameter. It is found that the temperature-dependent concurrence in the experiment can be well reproduced using the parameter value $\gamma^0_{PH} = 1\,\mathrm{ns}^{-1}$.

Photonic 2-qubit density matrix and concurrence

The quantum state tomography employed in the experiment is based on polarization-resolved two-time coincidence measurements. The obtained signals in this reconstruction scheme are theoretically described by two-time correlation functions which contain electric field operators at different times. Because the exciton and biexciton photons detected in the coincidence measurements stem from electronic transitions in the standard biexciton-exciton cascade, the electric field operators are proportional to QD transition operators $\hat{\sigma}_{H/V}$ =

$|G\rangle\langle X_{H/V}| + |X_{H/V}\rangle\langle XX|$. Consequently, the measured signals are theoretically calculated by evaluating correlation functions

$$G^{(2)}_{jk,\ell m}(t,\tau) = \left\langle \hat{\sigma}^\dagger_j(t)\hat{\sigma}^\dagger_k(t+\tau)\hat{\sigma}_m(t+\tau)\hat{\sigma}_\ell(t)\right\rangle = \mathrm{Tr}\left\{\hat{\sigma}^\dagger_k\hat{\sigma}_m\mathcal{P}_{t\to t+\tau}\left[\hat{\sigma}_\ell\mathcal{P}_{0\to t}\left[\hat{\rho}(0)\right]\hat{\sigma}^\dagger_j\right]\right\}, \quad (11)$$

with $\{j,k,\ell,m\} \in \{H,V\}$. Here, the time t is the (real) time of the first photon detection event and τ denotes the delay time until the second one occurs.

In the experimental setup, one considers all (real) times t until the QD returns to its ground state and accounts for all delay times τ that fall in a certain interval/time bin Δt. Thus, the (normalized) photonic 2-qubit density matrix ρ^{2q} is calculated from time-integrated correlation functions according to

$$\rho^{2q}_{jk,\ell m} = \frac{\overline{G}^{(2)}_{jk,\ell m}}{\mathrm{Tr}\left\{\overline{G}^{(2)}\right\}} \quad (12a)$$

$$\overline{G}^{(2)}_{jk,\ell m} = \int_0^\infty \mathrm{d}t \int_{t_0}^{t_0+\Delta t} \mathrm{d}\tau\, G^{(2)}_{jk,\ell m}(t,\tau), \quad (12b)$$

where t_0 is the starting time of the considered (delay) time bin.

Finally, the concurrence is directly calculated from the photonic 2-qubit density matrix according to[16]

$$C = \max\left\{0, \sqrt{\lambda_1} - \sqrt{\lambda_2} - \sqrt{\lambda_3} - \sqrt{\lambda_4}\right\}, \quad (13)$$

where $\lambda_j \geq \lambda_{j+1}$ are the (real and positive) eigenvalues of the 4×4-matrix

$$M = \rho^{2q}\, T\, (\rho^{2q})^*\, T \quad (14)$$

Here, $(\rho^{2q})^*$ denotes the complex conjugated 2-qubit density matrix, and T is the anti-diagonal matrix with elements $\{-1,1,1,-1\}$. Note that the concurrence also depends on t_0 and Δt.

For the simulations presented in Figure 1(e) of the Main Text, the time bin is always set to $\Delta t = 2\,\mathrm{ns}$. Additionally, in Figure 7 of the Supplementary Material, the evaluation is performed for three different time bins. In all these cases, delay times starting from $t_0 = 0$ are considered. In order to describe the time-filtering analysis in Figure 3 of the Main Text, numerical calculations with different starting times $t_0 = 0\,\mathrm{ps}, 350\,\mathrm{ps}, 700\,\mathrm{ps}, 1050\,\mathrm{ps}$ and a time bin of $\Delta t = 500\,\mathrm{ps}$ are performed.

References

(1) Trotta, R.; Martín-Sánchez, J.; Daruka, I.; Ortix, C.; Rastelli, A. Energy-Tunable Sources of Entangled Photons: A Viable Concept for Solid-State-Based Quantum Relays. *Phys. Rev. Lett.* **2015**, *114*, 150502.

(2) Huber, D.; Reindl, M.; Covre da Silva, S. F.; Schimpf, C.; Martín-Sánchez, J.; Huang, H.; Piredda, G.; Edlinger, J.; Rastelli, A.; Trotta, R. Strain-Tunable GaAs Quantum Dot: A Nearly Dephasing-Free Source of Entangled Photon Pairs on Demand. *Phys. Rev. Lett.* **2018**, *121*, 033902.

(3) Herklotz, A.; Plumhof, J. D.; Rastelli, A.; Schmidt, O. G.; Schultz, L.; Dörr, K. Electrical characterization of PMN–28%PT(001) crystals used as thin-film substrates. *J. Appl. Phys.* **2010**, *108*, 094101.

(4) James, D. F. V.; Kwiat, P. G.; Munro, W. J.; White, A. G. Measurement of qubits. *Phys. Rev. A* **2001**, *64*, 052312.

(5) Bahder, T. B. Eight-band k·p model of strained zinc-blende crystals. *Phys. Rev. B* **1990**, *41*, 11992–12001.

(6) Gawarecki, K.; Machnikowski, P.; Kuhn, T. Electron states in a double quantum dot with broken axial symmetry. *Phys. Rev. B* **2014**, *90*, 085437.

(7) Mielnik-Pyszczorski, A.; Gawarecki, K.; Gawełczyk, M.; Machnikowski, P. Dominant role of the shear strain induced admixture in spin-flip processes in self-assembled quantum dots. *Phys. Rev. B* **2018**, *97*, 245313.

(8) Gawełczyk, M.; Syperek, M.; Maryński, A.; Mrowiński, P.; Dusanowski, Ł.; Gawarecki, K.; Misiewicz, J.; Somers, A.; Reithmaier, J. P.; Höfling, S.; Sęk, G. Exciton lifetime and emission polarization dispersion in strongly in-plane asymmetric nanostructures. *Phys. Rev. B* **2017**, *96*, 245425.

(9) Andrzejewski, J.; Sęk, G.; O'Reilly, E.; Fiore, A.; Misiewicz, J. Eight-band k·p calculations of the composition contrast effect on the linear polarization properties of columnar quantum dots. *J. Appl. Phys.* **2010**, *107*, 073509.

(10) Thränhardt, A.; Ell, C.; Khitrova, G.; Gibbs, H. M. Relation between dipole moment and radiative lifetime in interface fluctuation quantum dots. *Phys. Rev. B* **2002**, *65*, 035327.

(11) Krzykowski, M.; Gawarecki, K.; Machnikowski, P. Hole spin-flip transitions in a self-assembled quantum dot. *Phys. Rev. B* **2020**, *102*, 205301.

(12) Woods, L. M.; Reinecke, T. L.; Kotlyar, R. Hole spin relaxation in quantum dots. *Phys. Rev. B* **2004**, *69*, 125330.

(13) Tighineanu, P.; Daveau, R.; Lee, E. H.; Song, J. D.; Stobbe, S.; Lodahl, P. Decay dynamics and exciton localization in large GaAs quantum dots grown by droplet epitaxy. *Phys. Rev. B* **2013**, *88*, 155320.

(14) Huber, D.; Lehner, B. U.; Csontosová, D.; Reindl, M.; Schuler, S.; Covre da Silva, S. F.; Klenovský, P.; Rastelli, A. Single-particle-picture breakdown in laterally weakly confining GaAs quantum dots. *Phys. Rev. B* **2019**, *100*, 235425.

(15) Lindblad, G. On the generators of quantum dynamical semigroups. *Commun. Math. Phys.* **1976**, *48*, 119.

(16) Wootters, W. K. Entanglement of Formation of an Arbitrary State of Two Qubits. *Phys. Rev. Lett.* **1998**, *80*, 2245.
